# Community Ophthalmology: Revisited

**DOI:** 10.4103/0970-0218.66893

**Published:** 2010-04

**Authors:** R Jose, A S Rathore, Sandeep Sachdeva

**Affiliations:** Directorate General of Health Services, Ministry of Health and Family Welfare, Nirman Bhawan, New Delhi-110 108, India

Visual impairment has immediate and long-term consequences in people of all age groups resulting in lost blind-person years, low educational and employment opportunities, poor economic gain for individual, families and societies and decreased quality of life.

According to WHO criteria, global estimate predict that there are 314 million people with visual impairment [45 million Blind (visual acuity <3/60) and 269 million due to Low Vision (visual acuity <6/18) due to eye diseases and refractive errors]. There has been a transition in usage of definition from ‘best corrected’ vision to ‘presenting’ vision in determining the extent of visual impairment. Best-corrected vision, refers to visual acuity obtained with the best possible refractive correction whereas presenting vision, indicate visual acuity obtained using currently available refractive correction, if any.(1)

Over the last 20 years, causes of blindness has changed both in proportion and actual numbers; however, cataract has still remained the major cause of blindness globally and more so in Asia. Globally, major causes are 17.6 million cataract [39%], 8 million refractive errors [18%], 4.5 million glaucoma [10%], 3.2 million age-related macular degeneration [7%], 1.9 million corneal scar [4%], 1.8 million diabetic retinopathy [4%], 1.3 million childhood [3%], 1.3 million trachoma [3%], 0.3 million onchocerciasis [0.7%] and 4.8 million due to other causes [11%].(2)

Available Indian estimates suggest that there are more than 12 million bilaterally blind persons in the country with visual acuity [VA] <6/60 in the better eye, of which nearly 7 million are with VA <3/60 in the better eye. National survey during 2001–04 indicated that prevalence of blindness stood at 1.1% and Rapid Assessment of Avoidable Blindness [RAAB] in 2006–07 showed that prevalence has come down to 1.0%. Main causes of blindness in the surveyed population indicated cataract [62.6%], refractive errors [19.7%], corneal blindness [0.9%], glaucoma [5.8%], surgical complication [1.2%], posterior capsular opacification [0.9%], posterior segment disorder [4.7%] and other causes [4.19%]. These people went blind due to various pathological causes but the reasons for which they continue to remain blind could be grouped into socio-demographic, nutritional, environmental and service delivery factors. This brief highlights the initiatives undertaken by Government of India in Eleventh Five-Year [2007–12] Plan and the role that can be played by Department of Community Medicine/PSM in eliminating avoidable causes of blindness in the light of increase scope and dimensions under the program.

## National Program for Control of Blindness

The focus of National Program for Control of Blindness [NPCB] since inception has been improving cataract surgical rate and coverage in the country. During the last thirty years, capacity of institutions, health personnel and community has witnessed a dramatic improvement in addressing such related issues. The program has been able to deliver effective eye care services through successful and vibrant Public Private Partnership [PPP], through decentralized mode under integrated State/District Health Societies of National Rural Health Mission [NRHM], a win-win situation for all stakeholders and parties. The word District Blindness Control Societies [DBCS] are no more in vogue. The year 2008 recorded ever-highest 5.8 million cataract surgeries with 94% intraocular lens [IOL] implantation at national level inspite of continuation of ban on ‘surgical camps’ in makeshift operation theaters to prevent post-operative infections. The program has been able to achieve huge quantitative gains without compromising quality and the momentum thus generated has paved way towards a sustainable blindness free society in near future.

## Eleventh Five-Year [2007-12] Plan Proposal

India being signatory to global call of International NGOs/WHO termed as ‘VISION 2020: The Right to Sight’ Initiative, Government of India is working towards making society free from all causes of avoidable [preventable plus treatable] blindness by the year 2020. Eye surgeons, medical officers and Mid-level Ophthalmic Personnel [ophthalmic assistant/optometrist/refractionist] in public health system along with non-governmental institutions are providing free eye care services to needy poor and private sectors for those who can afford. In the backdrop of developmental transition, impending epidemic of non-communicable diseases, containment of endemic/nutritional diseases like Trachoma and Vitamin A deficiency to focal/regional areas, a thrust has been injected in the program with a greater commitment to ensure better quality of vision/life to those affected. Newer/hitherto under covered eye disease entities are being addressed for the first time in the approved Eleventh Five-Year (2007–12) Plan of NPCB in a comprehensive manner, which got Cabinet approval for implementation in Oct’ 2008. The funding under the program for the Eleventh Plan has been enhanced to approx. $ 260 million USD three times the previous plan period, suggestive of high political commitment. India has received technical impetus and financial assistance from World Bank, WHO, DANIDA and other International NGOs for amelioration of blindness in the country but currently program is not dependent on any external funding.

## New Initiatives Under NPCB

Ongoing interventions under NPCB include cataract screening and management, eye screening of children in schools, prescription and dispensing of free spectacles, capacity building of health personnel, establishment of vision centres at primary and secondary level, strengthening activities related to eye donation/cornea collection and development of eye banks. Cataract surgeries are being performed using intraocular lens [IOL] implantation through modern technique of Small Incision Cataract Surgery [SICS] or Phacoemulsification both in public and private sector. Government of India [GoI] provides 100% financial assistance to all States/UTs for purchase of modern ophthalmic equipments, instrument, low cost good quality IOLs and consumable under the program. All State/District program officers have been instructed to display round the clock contact details of functional eye banks in all government in-patient facilities of the district in which they are currently located. Health personnel through in-service training program in a phase manner are being sensitized through a movie clip on procedure of eye donation so as to facilitate increase eye/cornea collection under Hospital Cornea Retrieval Program [HCRP]. Newer initiatives like Teleophthalmology, diseases other than cataract like Diabetic Retinopathy [DR], Glaucoma, Corneal Transplantation, Vitreo-retinal surgery, treatment of childhood blindness including Retinopathy of Prematurity [ROP] are being covered for the first time in the Eleventh Plan thus making eye care services comprehensive in nature. Financial assistance is also being provided to NGO institutions for carrying out above-mentioned activities.

Over the last few decades, multiple interjections of global advancement of knowledge, sharing of best practices and transfer of low cost technology, better classification of visually impaired, strong advocacy for fundamental right of blind persons and inclusive society has resulted in establishment of a distinct entity of ‘Low Vision’ amongst visually impaired. The person suffering from Low Vision are characterized by impairment of visual functioning even after medical, surgical treatment and/or ‘standard’ spectacles; however, such person have potential to use vision for planning and/or execution of a task. The causes of low vision in older people vary. In general, it could be due to glaucoma, diabetes, macular degeneration, hypertensive retinopathy or retinal detachment.(3)

People with ‘Low Vision’ are, in principle, capable of using their vision if given appropriate Low Vision Services including stimulating environmental/modification, assistive devices [e.g. Low Vision Devices (LVD) - high plus spectacles, magnifiers, telescopes, video-magnifiers, absorptive lenses, field expanding devices etc.], training, counseling and support; they do not necessarily need to use white canes or learn Braille. National Program for Control of Blindness has taken an initiative of increasing demand for low vision services through awareness generation activities, training of eye care teams on Low Vision, provision of selected LVD through identified Regional Institute of Ophthalmology [RIO], medical colleges and NGO institutions free of cost to BPL population.

## Social Mobilization

Worldwide people are living longer and birth rates are declining. From public health perspective, blindness usually affects early and later age spectrum of life and both ‘age cohorts’ are dependent on ‘others’ for taking them to health system in their problem amelioration. Childhood blindness remains a significant problem due to cumulative loss of blind-person years in young children though its magnitude is relatively small when compared to extent of blindness in older adults, as 82% of all blind persons are 50 years and above. Female/male prevalence ratio indicate that women are more likely to have visual impairment than men in every region of the world even after adjustment for age; the ratio range from 1.5 to 2.20.(4) Outreach screening, transportation and accompanying escort becomes an essential strategy and a challenge for reaching out to such diverse, disperse and dependent population. The fundamental issue under any program is social mobilization for advancement of health objectives and increasing demand and utilization of services. It is expected that health personnel including community link worker like ASHA, Anganwadi workers and ‘motivated’ members of civil society and PRI can play a critical role in this aspect. Village-wise blind register is a tool that facilitates in identification, recording, communication, referral and appropriate management of such cases. Funds are dispersed for purchasing ‘registers’ but due to various human factors these are neither maintained nor updated for action in most of the places. There are many developmental non-ophthalmic NGOs working in and with community that may not be currently associated with NPCB; a strategy to involve them at grassroot level is being devised for advancement of program objectives.

## Community Ophthalmology

Curative ophthalmology can make a perceptible impact in the society only in conjunction with community ophthalmology. Such activities include need assessment, planning, mobilizing level appropriate resources, fact finding surveys, outbreak investigation in ophthalmic practices, targeted interventions through screening camps in collaboration with department of Ophthalmology, operational research, clinical care, Vitamin-A supplement/rich food, complete vaccination [especially measles], training, ophthalmic surveillance; sensitization, counseling, motivation, ensuring compliance, referral, follow-up, rehabilitation of incurable blind, empowering community/individuals to utilize available government concessions/benefit for the welfare of blind; reducing myths and misconceptions, understanding and removing barriers for access to services, facilitating favorable environment for growth and development; local leadership and coordination amongst stakeholders under various governmental departments of health, social welfare, education, PRI and ICDS, establishment of intra and inter-linkages, information, education and communication activities [IEC], promotion of eye donation, improving efficient client movement and logical disposal within health facilities, feedback/reminders for action, monitoring, supervision and evaluation.

## Role and Contribution of Department of Community Medicine/PSM

Department of Community Medicine/PSM are undertaking/can uptake all the above activities in their area of jurisdiction and can strive to make it free from all causes of avoidable blindness. Since the scope and dimensions of the program has increased substantially in the Eleventh Plan, there is a huge potential and opportunity for department to participate and contribute. In an era of global recession where mantra of ‘multi-tasking’ has become a necessity rather than luxury, residents in the department of Community Medicine/PSM, in addition, can learn and enhance their ophthalmic screening and diagnostic skills. Howsoever small contribution it will be, still it will hold potential to benefit program at grassroot level.

## Sentinel Surveillance Units

Sentinel Surveillance Units [SSU] conceptualized under NPCB have been established under department of Community Medicine/PSM in collaboration with the Department of Ophthalmology in selective 25 medical colleges. These units are frontline soldiers of the program in carrying out surveillance activities. Financial assistance to SSU has been enhanced from INR 1.5 lakhs to the tune of INR 3 lakhs from this year onwards so as to re-vitalize and rejuvenate them. However, some of the challenges and concerns in these units are lack of local leadership, inadequate coordination between departments, poor communication, incomplete/under reporting and a few pro-active community interventions. Nevertheless, NPCB is marching ahead steadily, consolidating gains, expanding in underserved/difficult to reach areas, year after year with involvement of stakeholders at all level. Meaningful results have already started appearing and long-term and sustainable impact will be evident in times to come.

## References

[1] Resnikoff S, Pascolini D, Mariotti SP, Pokharel GP (2008). Global magnitude of visual impairment caused by uncorrected refractive errors in 2004. Bull World Health Organ.

[2] Foster A, Gilbert C, Johnson G (2008). Changing patterns in global blindness: 1988-2008. Community Eye Health J.

[3] Simon L (2008). Low vision and rehabilitation for older people: Integrating services into the health care system. Community Eye Health J.

[4] Resnikoff S, Pascolini D, Etya’ale D, Kocur I, Pararajasegaram R, Pokharel GP (2004). Global data on visual impairment in the year 2002. Bull World Health Organ.

